# The Antimicrobial and Cytotoxic Activity of Green‐Synthesized Silver Nanoparticle Extract of Marine Bacteria and Their 16S rRNA Identification and Characterization

**DOI:** 10.1155/bca/9040709

**Published:** 2026-09-20

**Authors:** Adel I. Alalawy, Haddad A. El Rabey, Fahad M. Almutairi, Amnah Obidan, Rehab F. Almassabi, Hayam A. Alwabsi, Ghena M. Al-Jahani, Nouran M. Salah

**Affiliations:** ^1^ Biochemistry Department, Faculty of Science, University of Tabuk, Tabuk, Saudi Arabia, ut.edu.sa; ^2^ Faculty of Biotechnology, University of Sadat City, Sadat City, Egypt, usc.edu.eg; ^3^ Nutrition Department, Faculty of Science, University of Tabuk, Tabuk, Saudi Arabia, ut.edu.sa; ^4^ Department of Microbiology and Immunology, Faculty of Pharmacy, Menoufia University, Shebin El-Kom, Menoufia, Egypt, menofia.edu.eg

**Keywords:** 16SR, AgNPs, bacteria, Candida, marine sponge

## Abstract

Marine bacteria represent a substantial underexplored reservoir of bioactive metabolites with significant biomedical potential. Through 16S rRNA gene sequencing, this study identified four bacterial isolates associated with the marine sponge *Callyspongia siphonella*. They were *Gammaproteobacterium*, *Idiomarina* sp., *Halomonas* sp., and *Oceanobacillus kapialis*. The isolates were used to synthesize silver nanoparticles (AgNPs) in an environmentally friendly way. Subsequently, the antibacterial and cytotoxic activities of these nanoparticles were evaluated. All biosynthesized AgNPs were very small (7.28–13.8 nm) and effective against common pathogens, with minimum inhibitory concentrations between 8 and 16 μg/mL. The nanoparticles exhibited no detectable inhibitory activity against *Candida albicans*, which means that they only worked against bacteria. Cytotoxicity assays showed that the four AgNP preparations had the same IC_50_ values (42–44 μg/mL) and that the cytotoxic activity against rat cancer cells increased with concentration. This indicates that the physicochemical characteristics of the nanoparticles, rather than their bacterial source, predominantly affected cytotoxic efficacy. Gram staining and microscopy‐based phenotypic characterization exhibited significant concordance with molecular identification, thereby reinforcing taxonomic classifications. These findings collectively highlight marine sponge‐associated bacteria as potential bioreactors for the sustainable synthesis of bioactive AgNPs with selective antimicrobial activity and moderate cytotoxic properties.

## 1. Introduction

Marine sponges are among the oldest and most evolutionarily diverse metazoan groups with complicated microbial communities that have become new sources of bioactive secondary metabolites used in many different biotechnological applications [1–3]. Marine sponges and the tiny organisms that symbiotically live on them have, for millions of years, founded a wide range of bacterial communities that produce bioactive compounds important for the host’s chemical defense systems [3]. These bacterial symbionts′ bioactive compounds, such as enzymes, can be used in the industry and may act as antioxidants or antimicrobial agents [2].

The genus *Callyspongia*, part of the family *Callyspongiidae*, has attracted significant interest because it possesses a large number of bacterial symbionts that can make a wide range of antibacterial chemicals [4, 5]. The marine sponge species *Callyspongia siphonella*, inhabiting tropical and subtropical waters, possesses a distinctive microbiome that remains inadequately explored for its prospective biotechnological applications [5].

Recent studies show that bacteria found in sponges, especially those from *Callyspongia* species, have strong antibacterial effects against medically important infections. This suggests they could be useful for identifying novel medications and harnessing biotechnology [6].

The growing antimicrobial resistance has increased the need to discover novel antimicrobial agents from various environments, such as marine environments, which are currently regarded as promising reservoirs of bioactive compounds [7]. If there is no solution for antibiotic resistance, by 2050, antimicrobial resistance could kill 10 million people every year, which is a risk factor for public health all over the world [8]. Traditional approaches to discovering new antibiotics have proven largely ineffective, necessitating the development of innovative methods that leverage the biosynthetic capabilities of marine micro‐organisms [9]. The biosynthesis of silver nanoparticles (AgNPs) using bacterial extracts has gained prominence due to their enhanced antimicrobial efficacy, reduced cytotoxicity compared to chemically synthesized counterparts, and environmentally sustainable production methods [10, 11].

AgNPs synthesized by biological processes are preferred for killing germs because they have unique physicochemical properties, such as a high surface area‐to‐volume ratio, controlled size distribution, and biomolecular capping agents that make them more stable and compatible with living organisms [12]. The green synthesis methods of bacterial metabolites, particularly proteins, enzymes, and secondary metabolites, reduce silver ions during biosynthesis while simultaneously contributing to the capping and stabilization of the resulting nanoparticles through producing safe nanoparticles that are more effective for treating diseases and have a lower environmental impact than traditional chemical and physical synthesis methods using nonharmful chemicals [10, 13].

Marine sponges have a lot of bacteria, and many of them, like *Gammaproteobacteria*, have strong bioactivity that protects the host. *Gammaproteobacteria* are cocci or rod‐shaped Gram‐negative [14]. They are often the most common type of bacteria in sponges that have antibacterial and even anticancer compounds that people used to think the sponge made itself [15]. *Idiomarina* is a type of *Gammaproteobacterium*, a slightly curved Gram‐negative rod, that grows in saltwater exhibiting antimicrobial and anticancer characteristics [16]. *Halomonas* spp. (also Gram‐negative, rod‐shaped, nonspore‐forming halophiles) release several secondary metabolites that can have a wide range of effects [17]. *Halomonas saccharevitans* extract also has anticancer activity [18]. *Oceanobacillus kapialis* is a Gram‐positive, strictly aerobic, spore‐forming, moderately halophilic bacterium belonging to the *Bacillaceae* family, with still undiscovered metabolites, while its relatives, like *Bacillus* and other *Bacillaceae*, make a lot of cyclic lipopeptides (surfactins, iturins, fengycins) that are very good at killing bacteria and cancer cells [19].

The current study seeks to examine the biotechnological capabilities of bacterial symbionts derived from *Ca. siphonella* for the biosynthesis of AgNPs exhibiting improved antimicrobial and cytotoxic characteristics.

## 2. Materials and Methods

### 2.1. Isolation and Culturing of Bacterial Symbionts From Marine Sponge

The bacterial samples were collected from the Red Sea marine sponge *Ca. siphonella*, Hurghada, Egypt. Samples were collected in sterile containers in seawater and transported to the lab to keep the microbes alive. Three times, the sponge’s exterior was washed with sterile artificial seawater in a laminar‐flow hood free of germs. This was done to get rid of any dirt and contaminants that were not tightly stuck. A sterile scalpel was used to cut out about one cubic centimeter (1 cm^3^) of the inner mesophyll tissue. This was done to lower the chance of bacteria getting on the surface. The tissue piece was mixed for 10 to 15 s with a sterile mortar and pestle and 10 mL of sterile normal saline. The homogenate was diluted from 10^−1^ to 10^−6^ in a dilution series using sterile saline solution. Aliquots of 100 μL from appropriate dilutions (10^−4^, 10^−5^, and 10^−^Marine Agar 2216 (Difco, USA)), half‐strength Nutrient Agar with 3% NaCl (w/v), and Zobell Marine Agar (HiMedia, India) were used for propagation. The plates were kept at 28°C for 7 to 30 days for growth. Cycloheximide and nystatin were added to the media to provide antibacterial and antifungal activity. The different colonies were picked from each other with different colors, shapes, and growth patterns. Finally, they were purified by streaking them on fresh media several times. Then, they were kept as pure cultures on Marine Agar slants at 4°C for later experiments [20, 21].

### 2.2. Preparation of Bacterial Extract for AgNP Synthesis

Each of the four purified bacterial isolates was cultured in 250‐mL Erlenmeyer flasks with 100 mL of Mueller–Hinton broth (HiMedia, India). The cultures were incubated in an orbital shaker incubator (Innova43, Germany) at 150 rpm and 28°C for 48 to 72 h to reach the late exponential growth phase and get the most biomass. Bacterial growth was monitored spectrophotometrically by measuring the optical density at 600 nm (OD_600_) at regular intervals from inoculation until the stationary phase. Uninoculated Mueller–Hinton broth was used as the blank. Accordingly, Isolates 9, 11, 15, and 16 were harvested after 48, 62, 48, and 72 h of incubation, respectively. After incubation, the bacterial cultures were centrifuged twice at 10,000 rpm (about 12,000 × g) for 15 min at 4°C using a refrigerated centrifuge (Hettich, Germany) to pellet the bacterial cells and get cell‐free supernatant. The clear supernatant, which contained extracellular metabolites, enzymes, and bioactive compounds, without disturbing the cell pellet. The cell‐free supernatant was then passed through a sterile 0.22‐μm syringe filter (Millipore, USA) to make sure that all bacterial cells were killed and that the sample was sterile. This sterile, cell‐free bacterial extract was used as a reducing and capping agent in the green synthesis of AgNPs [13, 22]. Following the completion of the biosynthesis reaction, the nanoparticle suspensions were centrifuged at 12,000 × g for 20 min at 4°C to pellet the formed AgNPs. The supernatants, containing soluble bacterial metabolites, unreacted silver species, and other non‐nanoparticulate components, were carefully discarded. The nanoparticle pellets were washed three times with sterile deionized water; after each washing step, the pellets were gently resuspended and centrifuged again under the same conditions. The final washed pellets were resuspended in sterile deionized water to the required concentration and used for subsequent characterization and biological assays. All purification steps were performed under aseptic conditions. Sterility of the filtered cell‐free bacterial supernatants was additionally verified by plating aliquots on nutrient agar and confirming the absence of microbial growth after incubation [22]. Bacterial cultures harvested at the time were used as a negative control to verify that the bacteria themselves did not contribute to the observed antimicrobial activity, whereas the synthesized nanoparticles did.

### 2.3. The Green Synthesis of AgNPs

AgNPs were synthesized using a biological reduction method. AgNPs‐9, AgNPs‐11, AgNPs‐15, and AgNPs‐16 were generated from the sterile cell–free supernatants of Isolates 9, 11, 15, and 16, respectively. For each bacterial isolate, 2.5 mL of sterile bacterial extract was added to 40 mL of warm deionized water (heated to 60°C) in a 100‐mL capped bottle on a magnetic stirrer (Thermo Scientific, USA). This mixture was stirred constantly at 300 rpm while 34 mg of silver nitrate (AgNO_3_; Sigma‐Aldrich, Germany) was added. To get the silver nitrate concentration to about 4 mM, deionized water was added until the total volume was 50 mL. The reaction mixture was kept in the dark at room temperature (25°C) for 24 to 48 h to allow the silver ions (Ag^+^) to form AgNPs (Ag^0^). Nanoparticle formation was confirmed by watching the solution change color from clear or pale yellow to dark brown. This proved that AgNPs had been made correctly. Control experiments with silver nitrate solution devoid of bacterial extract and bacterial extract without AgNO_3_ were concurrently conducted under uniform conditions to validate the involvement of bacterial metabolites in nanoparticle synthesis [23–25]. The four AgNP preparations were synthesized under identical reaction conditions and differed only in the bacterial isolate used to generate the cell‐free supernatant.

### 2.4. Characterization of AgNPs

#### 2.4.1. UV–Visible Spectroscopy

The formation of biosynthesized AgNPs was confirmed by UV–visible spectrophotometry. An AccuLab UVS‐85 double‐beam UV–vis. a spectrophotometer (USA) was used to record absorption spectra at room temperature at 200–800 nm. The nanoparticle suspension was diluted to the correct level, and deionized water was used as the blank reference. Researchers found that the characteristic surface plasmon resonance (SPR) peak of AgNPs is usually between 400 and 450 nm [26, 27].

#### 2.4.2. Transmission Electron Microscopy (TEM)

TEM analysis was performed on representative samples from each AgNP preparation to elucidate the ultrastructural morphology of the biosynthesized AgNPs. A 10‐μL sample of the nanoparticle suspension was diluted to 1 μg/mL. Then, it was carefully put on a carbon‐coated copper grid (300 mesh, Sigma‐Aldrich, Germany). The sample was left to dry in the open air at room temperature for 5 to 10 min to make sure that it stuck well to the grid surface. A TEM (JEOL JEM‐1010, Japan) was used to take TEM pictures at an accelerating voltage of 100 kV. High‐resolution TEM images at different magnifications were obtained (from 500,00x to 2,000,00x) to find out the size, shape, and spread of each nanoparticle and how they were stuck together [28, 29].

#### 2.4.3. Fourier Transform Infrared Spectroscopy (FTIR)

FTIR analysis was used to find the functional groups in biomolecules that are responsible for the reduction, capping, and stabilization of AgNPs. The AgNP suspension was spun at 12,000 rpm for 20 min, and the pellet was washed three times with deionized water to get rid of any biological material that was not bound. A freeze dryer (Labconco FreeZone, USA) was used to lyophilize the washed nanoparticles and turn them into a dry powder. Using a mortar and pestle, the dried AgNP powder (2–3 mg) was mixed well with potassium bromide (KBr) powder (200 mg). Then, a hydraulic press (PerkinElmer, USA) was used to press the mixture into a clear pellet. An FTIR spectrophotometer (PerkinElmer Spectrum Two, USA) was used to record FTIR spectra in the transmittance mode in the wavenumber range of 3500–500 cm^−1^. To find out what biomolecules make up the nanoparticle [30], the characteristic absorption bands are linked to different functional groups.

#### 2.4.4. Dynamic Light Scattering (DLS)

Particle hydrodynamic diameter and size distribution were determined by DLS using a NICOMP Nano ZLS particle size analyzer (Z3000 ZLS). Before measurement, the AgNP suspension was diluted tenfold with deionized water. An aliquot of 250 μL was then transferred into a low‐volume disposable cuvette. The sample was equilibrated at 20°C for 2 min before data acquisition [31].

### 2.5. Antimicrobial Susceptibility Testing

The antimicrobial efficacy of biosynthesized AgNPs was evaluated using the agar well diffusion (cup‐plate) method against standard pathogenic micro‐organisms from the American Type Culture Collection (ATCC): *Staphylococcus aureus* (ATCC 25923), *Escherichia coli* (ATCC 25922), *Pseudomonas aeruginosa* (ATCC 27853), *Klebsiella pneumoniae* (ATCC 700603), and *Candida albicans* (ATCC 90028). Bacterial and fungal inocula were prepared, ensuring that they matched the turbidity of the 0.5 McFarland standard. A standardized inoculum to evenly spread the surface of sterile Mueller–Hinton agar (MHA) plates for bacteria and Sabouraud dextrose agar (SDA) plates for *C. albicans*. After the inoculum had dried for 5 to 10 min, a sterile cork borer was used to make holes in the agar that were 6 mm wide. Then, 100 μL of the AgNP solution was put into each well. Two additional wells were filled with a mix of the two samples to see how well they worked together. On each plate, there were also negative controls, like wells filled with sterile bacterial extract and a silver nitrate solution at the same concentration as the synthesis. This was done to make sure that the nanoparticles were what caused the activity. The plates were kept at 37°C for a whole day. A caliper to measure the diameter (in millimeters) of the clear zone of inhibition around each well was used to see the antimicrobial activity [32].

### 2.6. Minimum Inhibitory Concentration (MIC) Determination

The broth microdilution method was used to find the MIC. It used sterile 96‐well flat‐bottom microtiter plates (Corning, USA). Three to five colonies were taken from overnight cultures on MHA and mixed with sterile saline to make a bacterial inoculum. The turbidity was adjusted to match the 0.5 McFarland standard (about 1.5 × 10^8^ CFU/mL) using a spectrophotometer at 600 nm (OD_600_ = 0.08–0.13). The standardized bacterial suspension was diluted 1:100 in Mueller–Hinton broth (Oxoid, UK) to get a final inoculum concentration of about 5 × 10^5^ CFU/mL in each test well. A twofold serial dilution of AgNPs in Mueller–Hinton broth was done, with concentrations ranging from 256 to 0.125 μg/mL. 100 μL of AgNP dilution and 100 μL of bacterial inoculum were added to each well. The last column on each plate had positive growth controls (bacteria without AgNPs), negative controls (sterile broth only), and sterility controls (AgNPs without bacteria). The plates were incubated at 37°C under aerobic conditions for 18 to 24 h. The MIC was the smallest amount of AgNPs that did not make the water cloudy or let bacteria grow [33, 34].

### 2.7. Minimum Bactericidal Concentration (MBC) Determination

After determining the MIC, the MBC was measured to determine whether the activity was bacteriostatic or bactericidal. A 10 μL sample from each well that did not show any visible growth at or above the MIC concentration was put on MHA plates (HiMedia, India). The plates were kept at 37°C for 24 h, and the number of colony‐forming units was counted. The MBC is the lowest concentration of AgNPs that resulted in a ≥ 99.9% reduction of the initial bacterial inoculum, as evidenced by the lack of colony growth or the presence of fewer than three colonies on the agar plate. To ensure reproducibility [34], all experiments were done three times.

The concentrations of the AgNP preparations were primarily expressed in μg/mL, corresponding to the concentrations applied in the biological assays. To facilitate comparison on a molar basis, the mass concentrations were additionally converted to silver‐equivalent molar concentrations using the atomic molar mass of silver (107.8682 g/mol). The molar concentration was calculated according to the following equation:

(1) CAgmol/L=Cμg/mL×10−3107.8682,

where *C*(μg/mL) represents the applied AgNP mass concentration. Accordingly, 1 μg/mL was equivalent to approximately 9.27 μM silver. These values are reported as silver‐equivalent molar concentrations and should not be interpreted as the molar concentration of individual nanoparticles.

### 2.8. Preliminary Screening of the Acute Cytotoxic Effects of AgNPs

A standard cytotoxicity assay was used to test the potential anticancer activity of the bacterial extract‐biosynthesized AgNPs against the Walker 256 (ATCC CCL‐38), a female *Rattus norvegicus* mammary gland carcinoma cell line obtained from the National Cancer Institute, Egypt. Authentication of cell lines was achieved through short tandem repeat (STR) profiling. Genotyping was conducted by a testing lab on a specified date using an STR kit. The STR profiles were compared to a reference database, resulting in a match of at least 95% for all tested cell lines. Additionally, testing for mycoplasma contamination confirmed that the cell lines were free of mycoplasma. Cancer cells were grown in sterile, flat‐bottom 96‐well tissue culture plates (Corning, USA) at a density of 1 × 10^4^ cells per well in Dulbecco’s modified Eagle’s medium (DMEM; Gibco, USA), with 10% fetal bovine serum and 1% penicillin–streptomycin. The plates were placed in a room at 5% CO_2_ and 37°C overnight to help the cells adhere. After the incubation period, the culture medium was replaced with 100 μL of fresh medium containing the synthesized AgNPs at the specified concentrations. After that, the cells and nanoparticles were put in standard incubation conditions for two hours. An inverted phase‐contrast microscope (Olympus CKX53, Japan) was used to count the number of living cells in each well at the beginning and end of the incubation period. A trypan blue exclusion dye (Sigma‐Aldrich, Germany) was used to tell the difference between cells that were alive and cells that were dead. The final cytotoxic effectiveness was measured by the percentage drop in the number of living cells after treatment compared to the untreated control cells. All assays were performed in triplicate, and the results were analyzed as mean ± standard deviation to ensure statistical validity [35–38].

### 2.9. Identification of Bacterial Isolates

#### 2.9.1. Gram Staining Technique

Preliminary identification of the purified bacterial isolates was performed using the Gram staining technique to differentiate Gram‐positive and Gram‐negative bacteria based on the cell wall structure. Freshly grown bacterial cultures were used to prepare thin smears on clean glass microscope slides. After air‐drying and heat‐fixing the smears, slides were sequentially treated with crystal violet (primary stain) for 1 min, followed by Gram’s iodine solution (mordant) for 1 min to fix the dye. The slides were then briefly rinsed with distilled water before being decolorized with 95% ethanol (decolorizer) for 5–10 s, a critical step distinguishing Gram‐negative bacteria (which readily lose the crystal violet‐iodine complex) from Gram‐positive bacteria (which retain it). Counterstaining was performed with safranin for 30 s, after which the slides were washed with water, blotted dry, and observed under oil immersion (1000x) using a bright‐field microscope (Olympus CX23, Japan). Bacteria that stayed purple were called Gram‐positive, and those that turned pink or red were called Gram‐negative. To make sure the results were accurate and could be repeated, the right controls and thin, evenly spread smears were used. This method is an important first step in figuring out what type of marine bacteria it is and putting it in the right group [39].

#### 2.9.2. 16S of rRNA Gene Sequencing

The HiPurA Bacterial Genomic DNA Purification Kit (HiMedia, India; Cat. No. MB505) was used to extract genomic DNA from the four bacterial isolates that had been purified. The manufacturer sent along instructions with the kit. To put it simply, bacterial cells from cultures that had been growing for a night were collected by spinning them at 10,000 rpm for 5 min. To break down the cell wall of Gram‐positive bacteria, the cell pellet was put back into a solution of 20 mg/mL lysozyme and kept at 37°C for 30 min. To help the cells break down proteins, 20 mg/mL of proteinase K was added, and the cells were lysed at 55°C for 30 min with the lysis buffer that came with it. The lysate was spun through HiElute Miniprep Spin Columns, and then, it was washed with prewash solution and wash solution to get rid of any dirt. To get pure genomic DNA, 100 μL of elution buffer (10 mM Tris–HCl, pH 8.5) was used. A NanoDrop 2000 spectrophotometer (Thermo Scientific, USA) was used to measure the absorbance at 260 nm and find out how concentrated and pure the extracted DNA was by calculating the A_260_/A_280_ ratio. DNA samples with A_260_/A_280_ ratios between 1.8 and 2.0 were considered suitable for further use.

The 16S rRNA gene (partial region) was amplified by polymerase chain reaction (PCR) using primers 714323 V4003 (H11) forward: AGAGTTTGATCATGGTCAG and (H10) reverse: GTATTACCGCGGCTGCTGG [39], which produces a PCR product for the 16S rRNA of about 500–550 bp long. The PCR amplification was done in 50 μL reaction volumes that had 25 μL of 2× PCR Master Mix (Thermo Scientific, USA), 1 μL of each primer (10 μM), 2 μL of template DNA (50–100 ng), and 21 μL of water that did not contain any nucleases. The first step in the PCR cycling conditions was denaturation at 95°C for 5 min. After that, there were 35 cycles of denaturation (30 s at 95°C), annealing (45 s at 55°C), and extension (90 s at 72°C). Lastly, there was an extension at 72°C for 10 min [40].

The PCR product was separated on a 1.5% agarose gel, stained with ethidium bromide, and visualized under UV light. A PCR purification kit from Qiagen (Germany) was used to purify the amplified products and then sequenced using Sanger sequencing technology. BioEdit software (Version 7.2) was used to analyze the 16S rRNA gene sequences. The Basic Local Alignment Search Tool (BLASTn) algorithm was used to compare the sequences with those of the National Center for Biotechnology Information (NCBI) GenBank database to see which species they belong to. Sequences showing ≥ 97% similarity to reference strains were classified as belonging to the same species. A phylogenetic relationship was plotted based on sequence homology to the most relevant sequences.

### 2.10. Phylogenetic Analysis

Using the EMBOSS suite (v6.6.0.0) on a Linux workstation, 16S rRNA sequences were put together into one consensus group. The reverse read was reverse‐complemented, and the reads were combined and checked for base conflicts before the consensus was made. To make a multiple sequence alignment, MUSCLE (v3.8.1551 by Robert C. Edgar) was used to align the assembled consensus and chosen reference sequences that were found in NCBI BLAST. The alignment was exported from the Linux environment and imported into MEGA (MEGA v12.1) for maximum‐likelihood (ML) tree inference (model selection and bootstrap support as specified in the figure legend).

ClustalW was used to line up partial 16S rRNA gene sequences to construct a phylogenetic analysis. The ML method in MEGA 12 used the Bayesian information criterion (BIC) to pick the best substitution model, which was Tamura 3‐parameter with a discrete gamma distribution [+G, 4 categories] and invariant sites [+I]. An extensive level‐5 Subtree‐Pruning‐Regrafting heuristic method was used to construct a ML phylogenetic tree. There was no branch‐swap filter, and the first tree was generated automatically using NJ/MP. The adaptive bootstrap analysis was applied with a standard error threshold of 5% to check the clade support values. The Newick tree file was exported from MEGA and then uploaded to the Interactive Tree Of Life (iTOL v7) web server for final labeling, annotation, and figure creation.

## 3. Results

### 3.1. Isolation of Bacterial Symbionts

**FIGURE 1:**
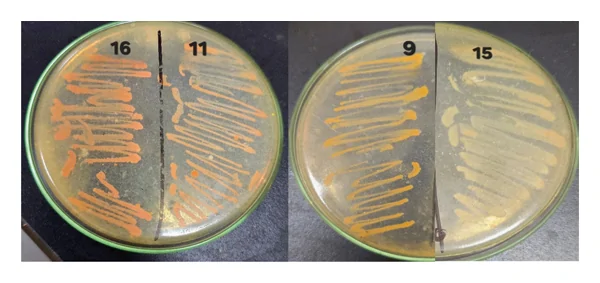
Culture of the four bacterial samples on marine agar.

Four marine bacterial symbionts were isolated from the marine sponge *Ca. siphonella*. The bacterial isolates were tested on marine agar and other types of media, such as *Actinomycetes* isolation agar. Figure 1 shows that Sample 16 was shiny orange, Sample 11 was light orange to brown, Sample 9 was golden yellow, and Sample 15 was beige with a smooth, mucoid look.

### 3.2. Green Synthesis and Characterization of AgNPs

**FIGURE 2:**
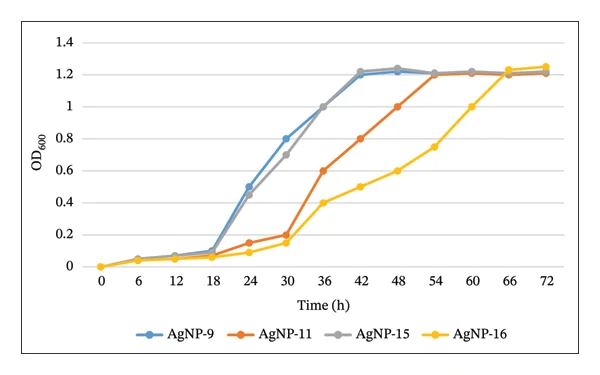
Growth curves of Marine Bacterial Isolates 9, 11, 15, and 16 cultured in Mueller–Hinton broth at 28°C with shaking at 150 rpm. Bacterial growth was monitored by measuring the optical density at 600 nm (OD_600_) at defined time intervals. The selected culture‐harvest times of 48, 62, 48, and 72 h, respectively, for the preparation of the cell‐free bacterial supernatants used in silver nanoparticle synthesis.

**FIGURE 3:**
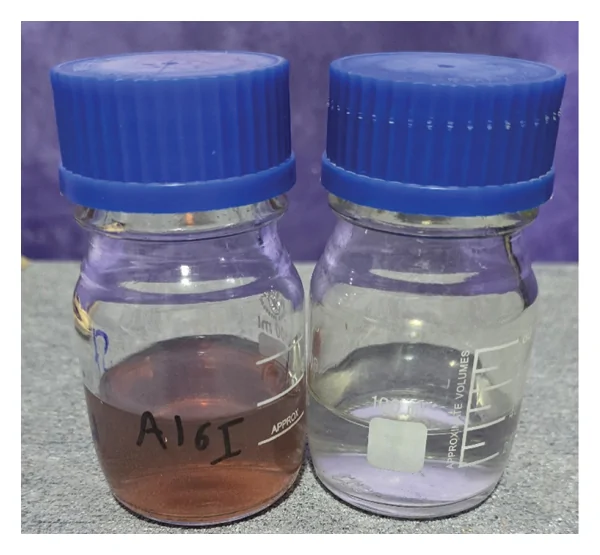
The left bottle turns brown, indicating nanoparticle formation. The right bottle’s silver nitrate stays colorless.

Bacterial cultures were harvested according to the late stationary phase of each isolate by monitoring bacterial growth at OD_600_, as shown in Figure 2. The biosynthesis of AgNPs was successfully achieved using the cell‐free supernatant from four marine bacterial isolates as efficient bioreducing and capping agents. When aqueous silver nitrate (AgNO_3_) was first added to the bacterial extracts, the reaction mixture slowly changed color. This was the first sign that nanoparticles were forming. The color changed from pale yellow to light brown in less than an hour when it was kept in the dark at room temperature. The new AgNPs’ SPR is what causes the color change. This means that the metabolites in the bacterial extract have successfully turned the silver ions (Ag^+^) into metallic silver (Ag^0^). The reaction was given a full 24 h to happen. The suspension’s color got darker and darker during this time, until it was a dark brown. This meant that nanoparticles were still forming and building up (Figure 3). This observation suggests that the bioreduction process was transient, marked by a swift initial phase followed by a gradual and steady growth phase. The control flasks containing solely silver nitrate exhibited no color change, indicating that the bioreduction was solely attributable to components of the bacterial cell‐free supernatant. The final dark brown colloidal suspension stayed for several weeks with no signs of precipitation. This showed even more that the biomolecules in the extract had successfully capped and stabilized the newly formed nanoparticles.

### 3.3. Characterization of AgNPs

#### 3.3.1. UV–Vis Spectroscopy

**FIGURE 4:**
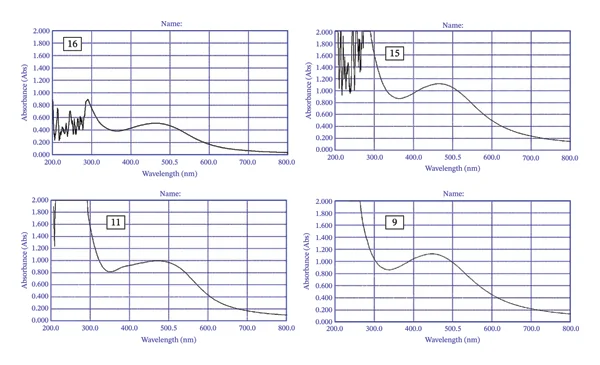
UV–vis spectroscopy curves for the four AgNP samples.

UV–visible spectroscopy was used to look at the absorption spectra of all four AgNP samples across the wavelength range of 200–800 nm. This helped us learn about the optical properties and confirm that AgNPs had formed (Figure 4). All four biosynthesized AgNP preparations displayed distinctive absorption profiles indicative of successful nanoparticle synthesis. The UV–vis spectra showed strong absorption peaks at about 460 nm, which is the same as the unique SPR peak that metallic AgNPs have. The four samples showed different levels of absorption intensity at this wavelength. This is because the four bacterial isolate‐derived preparations had different concentrations of nanoparticles and optical properties.

#### 3.3.2. FTIR

**FIGURE 5:**
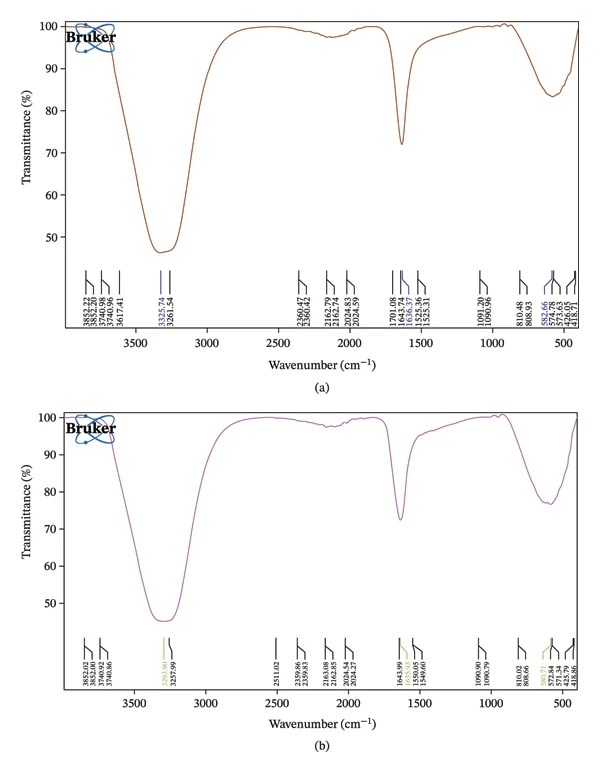
Curves for FTIR results of AgNPs and negative control of AgNO_3_, (a); FTIR for AgNO_3_, (b); FTIR for AgNPs.

FTIR spectroscopy was employed to identify functional groups and confirm the synthesis of AgNPs through bio‐reduction. The FTIR spectra of the blank control (a silver nitrate solution without a reducing agent) (Figure 5a) were compared to the biosynthesized AgNP sample (Figure 5b) over the whole spectral range (400–4000 cm^−1^) to make sure that AgNP synthesis worked and to find proof of the reduction process.

The FTIR spectrum of the blank control (AgNO_3_ solution) exhibited unique absorption bands associated with the silver salt precursor. There were sharp peaks at 3652, 3632, 3617, 3510, and 3490 cm^−1^ in the O–H/N–H stretching region, and broad bands at 3325 and 3261 cm^−1^. The vibrations of water and hydroxyl groups in the silver nitrate solution caused these peaks and bands. The carbonyl and functional group regions had strong peaks at 1701, 1684, 1636, 1538, 1525, and 1355 cm^−1^. The absorption was strongest at 1091 cm^−1^. This demonstrated that the C–O and C–H vibrations were due to interactions with the solvent. In the important low‐wavenumber range (400–700 cm^−1^), absorption bands showed up at 542, 508, 505, and 418 cm^−1^. The vibrations of the silver salt and solvent matrix caused these bands, but they did not have the typical Ag–O and Ag–O–Ag stretching signatures.

The FTIR spectrum of the biosynthesized AgNPs exhibited significant variations, clearly indicating the formation of AgNPs and the successful bioreduction of Ag+ ions. The peak pattern in the low‐wavenumber range (400–700 cm^−1^) was very different, with absorption bands at 520, 514, 479, and 413 cm^−1^. The strong absorption peaks between 500 and 650 cm^−1^ show Ag–O and Ag–O–Ag stretching vibrations. These are signs of AgNPs and were not present in the AgNO_3_ blank control. There was also a big bathochromic shift (red shift) in the fingerprint region, with peaks moving from 860 and 868 cm^−1^ (blank) to 810 and 806 cm^−1^ (AgNP sample). This is a drop of about 50 cm^−1^ in frequency. This peak shift shows that the nanoparticles and the capping biomolecules from the reducing agent are changing shape and interacting with each other. The carbonyl and amide regions (1000–1800 cm^−1^) showed different absorption patterns. The AgNP sample had peaks at 1643, 1598, 1550, and 1500 cm^−1^, while the blank had peaks at 1701, 1684, 1636, 1538, 1525, and 1355 cm^−1^. This means that functional groups in the plant extract helped both reduce Ag^+^ ions to Ag^0^ and cap the nanoparticles formed via surface coordination. The FTIR results in Table 1 show that biological reduction worked to synthesize AgNPs. The results also show that the process worked because it made Ag–O bonds, which are typical of AgNPs. The FTIR spectra of the four AgNP preparations showed comparable profiles, with no meaningful differences in the positions of the principal absorption bands. Therefore, one representative AgNP spectrum was presented together with the AgNO_3_ blank to avoid redundant presentation of essentially identical spectra, as shown in Figure 5.

**TABLE 1:** Comparative FTIR spectroscopic analysis of AgNO_3_ blank control and silver nanoparticle sample.

Spectral region (cm^−1^)	Functional group assignment	AgNO_3_ blank (cm^−1^)	AgNP sample (cm^−1^)	Spectroscopic significance
3700–3200	O–H stretching, H‐bonded water (aqueous solutions)	3652, 3632, 3617, 3510, 3490; broad: 3325, 3261	3652, 3632, 3510; broad: 3280, 3257	Similar in both; water/OH groups from aqueous salt solution
2163–2062	C≡N stretching or complexed functional groups	2162, 2024	2163, 2062	Minor shifts; possible coordination with metal
1701–1355	C=O, C=C, and amide bands (biomolecules from reducing agent)	1701, 1684, 1636, 1538, 1525, 1355	1643, 1598, 1550, 1500	Intensity reduction and frequency shifts in AgNP sample indicate biomolecule involvement in reduction and capping
1400–1000	C–O stretching (solvent, alcohols, ethers)	1091	1090	Similar patterns; reflects solvent composition
868–806	C–H out‐of‐plane bending (organic compounds)	860, 868	810, 806	Red shift of ∼50 cm^−1^ indicates nanoparticle formation and biomolecule–AgNP interaction
500–650^∗^	Ag–O and Ag–O–Ag stretching (diagnostic for AgNPs)	542, 508, 505, 418	520, 514, 479, 413	New peaks in the AgNP sample absent in AgNO_3_ blank: Direct evidence of Ag–O and Ag–O–Ag bonds forming silver nanoparticles

^∗^The emergence of NEW peaks in the 500–650 cm^−1^ range in the AgNP sample, which are NOT present in the AgNO_3_ blank, is the clearest sign that silver nanoparticles have formed. These peaks are diagnostic Ag–O and Ag–O–Ag stretching vibrations that are typical of silver nanoparticles.

#### 3.3.3. TEM

**FIGURE 6:**
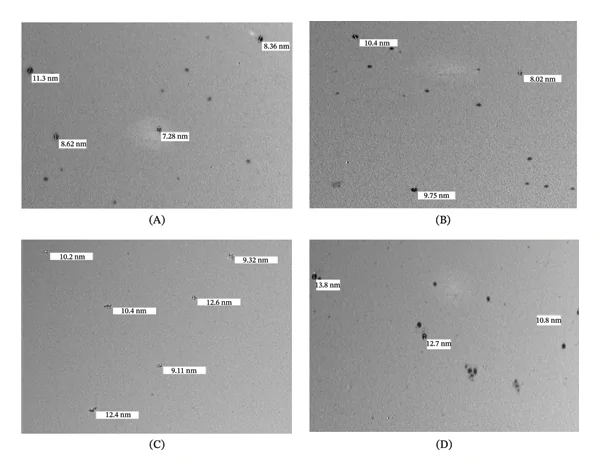
AgNPs obtained from four marine bacterial samples (15, 16, 11, 9) observed by TEM.

TEM analysis yielded high‐resolution images illustrating the morphological attributes and size distributions of the biosynthesized AgNPs derived from all four marine bacterial isolates (Figure 6). The TEM images showed that all of the AgNP samples (A, B, C, and D, which correspond to AgNPs‐15, AgNPs‐16, AgNPs‐9, and AgNPs‐11) mostly had spherical shapes and particle size distributions that were fairly uniform. The AgNPs‐15 sample (Figure 6A) had the smallest spherical nanoparticles that were evenly spaced apart. The particles were about 8.89 ± 1.56 nm in size on average. The shape of AgNPs‐16 (Figure 6B) was the same as the shape of the other particles. The particles were round and spread out evenly over the grid that was covered in carbon. The average size was 9.39 ± 1.05 nm. AgNPs‐9 (Figure 6C) had the widest range of sizes, with an average size of 10.68 ± 1.47 nm. On the other hand, the AgNPs‐11 sample (Figure 6D) had the biggest particles, with an average diameter of 12.43 ± 1.50 nm. The particles in all of the samples were between 7.28 and 13.8 nm in size.

#### 3.3.4. DLS

**FIGURE 7:**
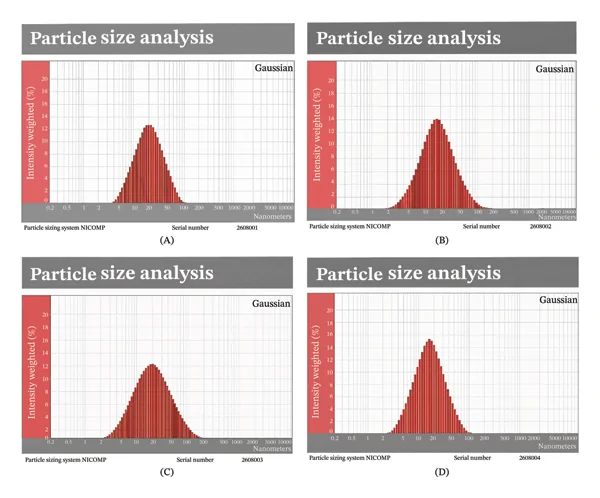
Intensity‐weighted particle size distributions of the biosynthesized silver nanoparticles determined by dynamic light scattering (DLS): (A) AgNPs‐15, with a modal hydrodynamic diameter of approximately 20 nm; (B) AgNPs‐16, approximately 18 nm; (C) AgNPs‐9, approximately 19 nm; and (D) AgNPs‐11, approximately 17 nm. The corresponding maximum peak intensities were approximately 13%, 14%, 12%, and 15%, respectively.

DLS analysis revealed single, approximately bell‐shaped intensity‐weighted particle size distributions for all four AgNP preparations as shown in Figure 7 (A, B, C, and D, which correspond to AgNPs‐15, AgNPs‐16, AgNPs‐9, and AgNPs‐11), indicating the predominance of one particle population in each sample. AgNPs‐11 exhibited the smallest modal hydrodynamic diameter, with a distribution maximum at approximately 17 nm and a peak intensity of 15%. The modal diameters of AgNPs‐9 and AgNPs‐16 were approximately 19 and 18 nm, with maximum intensities of 12% and 14%, respectively, whereas AgNPs‐15 displayed the largest modal diameter at approximately 20 nm, with a peak intensity of 13%. Among the examined preparations, AgNPs‐9 showed a comparatively broader size distribution, while AgNPs‐11 exhibited a sharper and more narrowly defined peak. No distinct secondary populations were observed, suggesting a limited contribution from larger particle aggregates under the measurement conditions.

### 3.4. Antimicrobial Activity of Biosynthesized AgNPs

**FIGURE 8:**
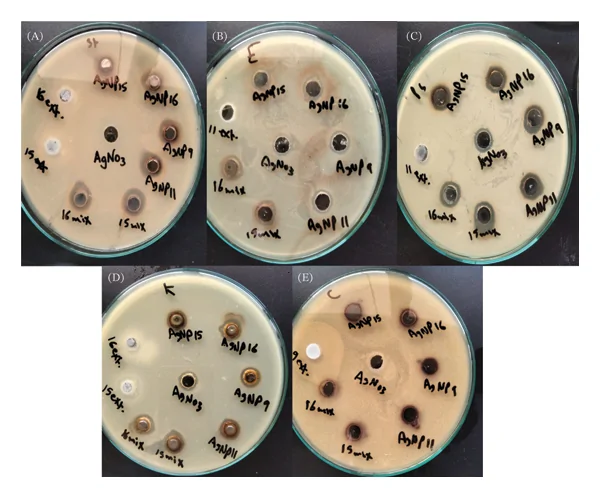
Antimicrobial activity of silver nanoparticle samples (AgNPs 9, AgNPs 11, AgNPs 15, and AgNPs 16) and mixtures between them (15 mix and 16 mix) was observed by inhibition zones using the well diffusion method. Negative controls of bacterial cultures show no inhibition zones, while AgNO_3_ control shows a small inhibition zone in some plates. All AgNP samples show inhibition zones against the four types of bacteria in different sizes in (A): *S. aureus*, (B): *E. coli*, (C): *Ps. aeruginosa*, (D): *K. pneumonia*. (E): *Candida albicans* shows no antifungal activity.

The agar well diffusion method demonstrated that all four biosynthesized AgNP samples exhibited significant antimicrobial activity against the tested bacterial pathogens and the fungal pathogen *C. albicans* (Figure 8). Each sample of AgNPs exhibited consistent and reproducible inhibition zones against *S. aureus, E. coli, K. pneumoniae,* and *P. aeruginosa*. The inhibition zones had diameters ranging from 14 to 17 mm (Table 2). The four nanoparticles derived from bacterial isolates (AgNPs‐15, AgNPs‐16, AgNPs‐9, and AgNPs‐11) exhibited varying levels of antimicrobial effectiveness, although they were generally similar. AgNPs‐16 and AgNPs‐11 had inhibition zones that were a little bigger (about 17 mm) against Ps. aeruginosa than the other isolates. *Ps. aeruginosa* demonstrated the highest susceptibility to AgNPs among all samples, suggesting enhanced nanoparticle penetration and bactericidal effectiveness against this Gram‐negative pathogen. In contrast, all tested AgNP samples demonstrated no antimicrobial activity against *C. albicans*, indicating that the structural and compositional attributes of the fungal cell wall may confer resistance to the synthesized nanoparticles.

**TABLE 2:** The agar well diffusion method was used to measure the inhibition zones (in millimeters) of biosynthesized silver nanoparticles and their combinations against standard pathogenic micro‐organisms.

Sample	*S. aureus* (mm)	*E. coli* (mm)	*K. pneumoniae* (mm)	*Ps. aeruginosa* (mm)	*C. albicans* (mm)
AgNPs‐15	14.67 ± 0.33	15 ± 0.0	14.33 ± 0.33	15.83 ± 0.17	—
AgNPs‐16	14.5 ± 0.29	15.33 ± 0.33	14.5 ± 0.29	16.83 ± 0.17	—
AgNPs‐9	14.17 ± 0.17	15.33 ± 0.33	14.17 ± 0.17	16.20 ± 0.19	—
AgNPs‐11	14.5 ± 0.29	15.07 ± 0.07	14.17 ± 0.17	17 ± 0.0	—
AgNPs‐I5‐9 mix	14 ± 0.0	15.17 ± 0.17	14 ± 0.0	16.17 ± 0.17	—
AgNPs‐I6‐11 mix	15.17 ± 0.17	15.17 ± 0.17	14.20 ± 0.19	16 ± 0.0	—
Control‐15	—	—	—	—	—
Control‐16	—	—	—	—	—
Control‐9	—	—	—	—	—
Control‐11	—	—	—	—	—
Control (AgNO_3_)	—	—	12.67 ± 0.33	13 ± 0.0	—

*Note:* Values show the average diameters of the inhibitory zones and the mean zones of inhibition; a dash means that no inhibitory zone was seen.

The combination samples (AgNPs‐I5‐9 mix and AgNPs‐I6‐11 mix) exhibited inhibitory activity that was either comparable to or slightly inferior to that of their individual constituents, indicating an absence of synergistic effects among these nanoparticle combinations under the evaluated conditions. All negative controls for the bacterial extracts (Control‐15, Control‐16, Control‐9, and Control‐11) showed no detectable inhibitory zones against any of the tested micro‐organisms. This clearly shows that the antimicrobial activity was only caused by the biosynthesized AgNPs and not by leftover bacterial metabolites. The silver nitrate control (control AgNO_3_) showed very little activity against *K. pneumoniae* and *Ps. aeruginosa* (about 13 mm), which is expected because silver ions are toxic on their own. However, this activity was much lower than that of the fully formed nanoparticles. The consistency of individual AgNP samples across triplicate assays, coupled with their clear differentiation from controls, confirms the reproducibility and reliability of the synthesis process and the antimicrobial evaluation methodology.

### 3.5. MIC of AgNPs

To find the MIC for each biosynthesized AgNP sample against four standard bacteria, a broth microdilution was used in 96‐well plates (Supporting Figure 1). This was a way to measure the nanoparticles′ antimicrobial activity in numbers. The MIC values in Table 3 showed that the AgNPs tested exhibited very different levels of antimicrobial activity. The AgNPs‐15 and AgNPs‐16 samples had the best broad‐spectrum activity. On the other hand, samples AgNPs‐11 and AgNPs‐9 had higher MICs, which means that they were not as effective as AgNPs‐15 and AgNPs‐16. The four nanoparticle samples had strong inhibitory activity against *E. coli*, with a MIC of about 8 μg/mL for all of them.

**TABLE 3:** Mean minimum inhibitory concentrations (MICs) ± SE of silver nanoparticle samples (from triplicates).

AgNP sample	*S. aureus* (μg/mL)	*S. aureus* (μM)	*E. coli* (μg/mL)	*E. coli* (μM)	*K. pneumoniae* (μg/mL)	*K. pneumoniae* (μM)	*Ps. aeruginosa* (μg/mL)	*Ps. aeruginosa* (μM)
15	8 ± 0.0	74.16 ± 0.00	10.67 ± 3.08	98.92 ± 28.55	8 ± 0.0	74.16 ± 0.00	13.33 ± 2.31	123.58 ± 21.42
16	13.33 ± 2.31	123.58 ± 21.42	8 ± 0.0	74.16 ± 0.00	10.67 ± 3.08	98.92 ± 28.55	8 ± 0.0	74.16 ± 0.00
9	10.67 ± 3.08	98.92 ± 28.55	8 ± 0.0	74.16 ± 0.00	16 ± 0.0	148.33 ± 0.00	10.67 ± 3.08	98.92 ± 28.55
11	10.67 ± 3.08	98.92 ± 28.55	10.67 ± 3.08	98.92 ± 28.55	8 ± 0.0	74.16 ± 0.00	13.33 ± 2.31	123.58 ± 21.42

### 3.6. MBC Determination

Following the determination of MICs, the bactericidal activity of the biosynthesized AgNPs was quantified by assessing their MBCs, as summarized in Table 4 and Supporting Figure 2. The four AgNP samples demonstrated variable but generally comparable bactericidal efficacy across the tested bacterial pathogens, with substantial sample‐dependent differences in MBC values. Against *S. aureus*, Samples 15 and 16 exhibited the most potent bactericidal activity with MBC values of 21.33 ± 5.33 μg/mL, while Samples 11 and 9 required higher concentrations of 32 ± 0.0 μg/mL, indicating a twofold reduction in efficacy for the latter pair. For *E. coli*, the bactericidal concentrations ranged from 16 ± 0.0 μg/mL for Samples 16 and 11 to significantly higher values of 26.67 ± 5.33 and 32 ± 0.0 μg/mL for Samples 15 and 9, respectively. Against *K. pneumoniae*, pronounced heterogeneity in MBC values was observed, with Samples 15 and 16 requiring the lowest bactericidal concentrations of 16 ± 0.0 μg/mL, while Samples 11 and 9 demonstrated progressively higher requirements of 21.33 ± 0.0 and 53.33 ± 10.67 μg/mL, respectively, with Sample 9 showing the most substantial increase, suggesting diminished bactericidal efficacy against this pathogen.

**TABLE 4:** Mean minimum bactericidal concentrations (MBCs) of silver nanoparticle samples (from triplicates).

AgNP sample	*S. aureus* (μg/mL)	*S. aureus* (μM)	*E. coli* (μg/mL)	*E. coli* (μM)	*K. pneumoniae* (μg/mL)	*K. pneumoniae* (μM)	*Ps. aeruginosa* (μg/mL)	*Ps. aeruginosa* (μM)
15	21.33 ± 5.33	197.74 ± 49.41	26.67 ± 5.33	247.25 ± 49.41	16 ± 0.0	148.33 ± 0.00	32 ± 0.0	296.66 ± 0.00
16	21.33 ± 5.33	197.74 ± 49.41	16 ± 0.0	148.33 ± 0.00	21.33 ± 5.33	197.74 ± 49.41	26.67 ± 5.33	247.25 ± 49.41
9	32 ± 0.0	296.66 ± 0.00	32 ± 0.0	296.66 ± 0.00	53.33 ± 10.67	494.40 ± 98.92	32 ± 0.0	296.66 ± 0.00
11	32 ± 0.0	296.66 ± 0.00	21.33 ± 5.33	197.74 ± 49.41	21.33 ± 5.33	197.74 ± 49.41	26.67 ± 5.33	247.25 ± 49.41

For *Ps. aeruginosa*, all four samples exhibited uniform bactericidal efficacy with MBC values clustering around 26.67–32 ± 0.0–5.33 μg/mL, with samples 15 and 9 requiring 32 ± 0.0 μg/mL, while Samples 16 and 11 demonstrated slightly lower requirements of 26.67 ± 5.33 μg/mL. Critically, the MBC values were consistently 2.0 to 4 times higher than their corresponding MIC values across all samples and bacterial species, confirming that the antimicrobial effects of these nanoparticles are predominantly bactericidal rather than bacteriostatic.

### 3.7. Preliminary Screening of the Acute Cytotoxic Effects of AgNPs

**FIGURE 9:**
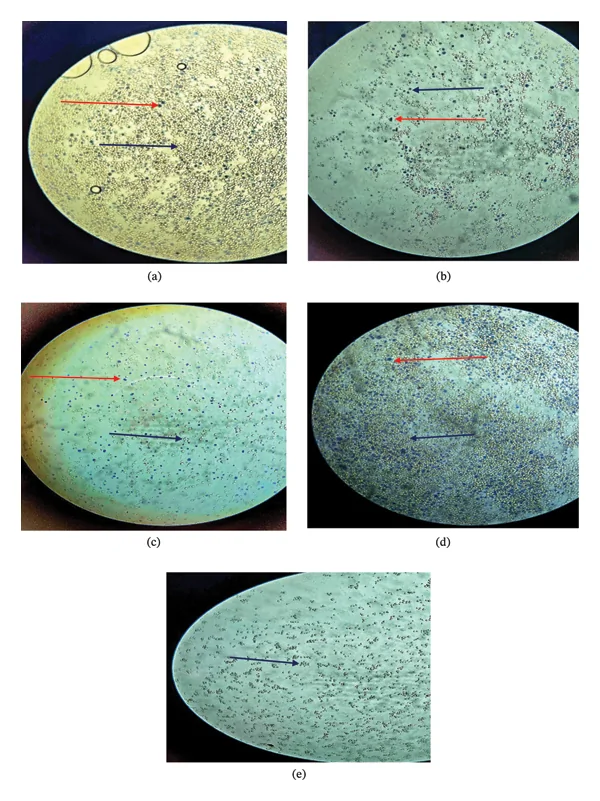
Microscopic screening of cytotoxic activity of four nanoparticle samples; dead cells appear in blue (red arrow), and live cells appear colorless (blue arrow). (a); AgNPs‐15, (b); AgNPs‐16, (c); AgNPs‐11, (d); AgNPs‐9, (e); negative control.

The initial cytotoxic efficacy of the biosynthesized AgNPs was evaluated using a trypan blue exclusion assay following a 2‐h incubation with rat cancer cells (Figure 9). Microscopic examination revealed substantial cytotoxic effects in all four AgNP samples (AgNPs‐15, AgNPs‐16, AgNPs‐11, and AgNPs‐9), as evidenced by the prevalence of blue‐stained cells, indicative of disrupted membrane integrity and cellular death (Figure 9a–d). The blue‐stained dead cells looked like separate blue balls all over the microscopic field. The living cells, on the other hand, did not change color. After being exposed to all four nanoparticle samples for two hours, a lot of cells died in all treatment groups. In stark contrast, the negative control groups—including untreated cancer cells, cancer cells treated with DMSO vehicle control, and cancer cells exposed to silver nitrate alone—exhibited minimal to no blue‐stained cells, confirming the viability of cells under control conditions and demonstrating that the observed cytotoxicity was specifically attributable to the biosynthesized AgNPs rather than the solvent or free silver ions (Figure 9e). Following two hours of exposure, all four AgNP preparations produced concentration‐dependent reductions in viable cells, as assessed by trypan blue exclusion. Because trypan blue primarily detects the loss of plasma‐membrane integrity, these findings indicate acute cytotoxic effects under the tested conditions but do not establish apoptosis, long‐term antiproliferative activity, or selective anticancer efficacy.

### 3.8. The Cytotoxic Efficacy of Biosynthesized AgNPs Against Rat Cancer Cells

**FIGURE 10:**
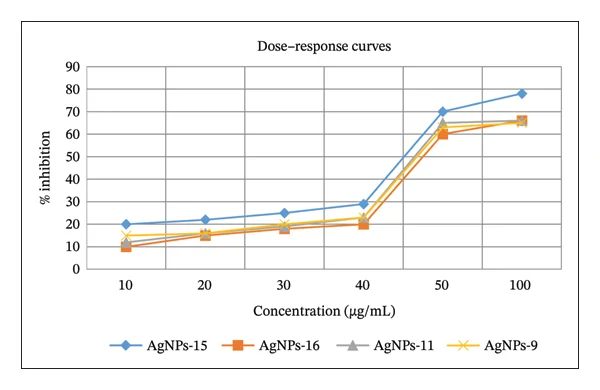
Dose–response curves of four nanoparticle samples.

A dose–response analysis quantitatively assessed the efficacy of biosynthesized AgNPs in eliminating rat cancer cells. Table 5 and Figure 10 show the mean percentage of inhibition from three experiments. The four AgNP samples (AgNPs‐15, AgNPs‐16, AgNPs‐11, and AgNPs‐9) were cytotoxic, but the level of toxicity depended on the concentration. The higher the concentration of nanoparticles, the higher the percentage of inhibition, which ranged from 10 to 100 μg/mL. There was a little bit of inhibition at the lowest concentration (10 μg/mL), ranging from 10% (AgNPs‐16) to 20% (AgNPs‐15). In all of the samples, the cytotoxic activity changed a lot between 40 and 50 μg/mL. This was a very important time for change.

**TABLE 5:** Mean %inhibition of triplicate results for AgNP concentrations.

AgNP concentration (μg/mL)	AgNPs‐15	AgNPs‐16	AgNPs‐11	AgNPs‐9
10	20 ± 1.16	10 ± 0.0	12 ± 0.58	15 ± 0.0
20	22 ± 1.16	15 ± 0.58	16 ± 1.16	16 ± 0.58
30	25 ± 1.73	18 ± 0.58	19 ± 1.73	20 ± 1.16
40	29 ± 1.15	20 ± 0.0	23 ± 1.73	23 ± 1.16
50	70 ± 2.89	60 ± 1.16	65 ± 2.89	63 ± 1.73
100	78 ± 1.15	66 ± 1.16	66 ± 1.15	65 ± 1.73

The IC50 values, which are the concentrations that stop half of the activity, were found using the dose–response curves. These numbers showed that all four nanoparticle samples were very similar in how harmful they were to cells. The IC50 values were 44 μg/mL for AgNPs‐15, 44 μg/mL for AgNPs‐16, 42 μg/mL for AgNPs‐11, and 43 μg/mL for AgNPs‐9. The IC50 values are very similar, ranging from 42 to 44 μg/mL. This means that all four AgNP formulations made from marine bacteria have the same effect on cancer, even though they came from different types of bacteria.

### 3.9. Identification of Bacterial Isolates

#### 3.9.1. Microscopic Identification

A preliminary morphological and differential characterization of the four marine bacterial isolates was done using Gram staining and a microscope (Supporting Figure 3). The Gram staining results showed that the isolated bacteria have different cell walls. Isolates 15 and 11 exhibited characteristics indicative of Gram‐negative bacteria, including cells displaying pink or red coloration, which signifies thin peptidoglycan layers and outer membrane structures. Isolates 9 and 16, on the other hand, showed Gram‐positive morphology and stained purple/violet, which means they have thick peptidoglycan cell walls and no outer membrane. These isolates predominantly exhibited slightly ovoid to rod‐shaped morphologies, characteristic of coccobacilli or bacilli. These morphological observations were consistent and reproducible across various microscopic fields and replicates, establishing a foundational basis for subsequent molecular identification through 16S rRNA sequencing, which would yield definitive species‐level classification and phylogenetic positioning of these marine sponge‐associated bacterial symbionts.

#### 3.9.2. Molecular Identification by 16s rRNA

**FIGURE 11:**
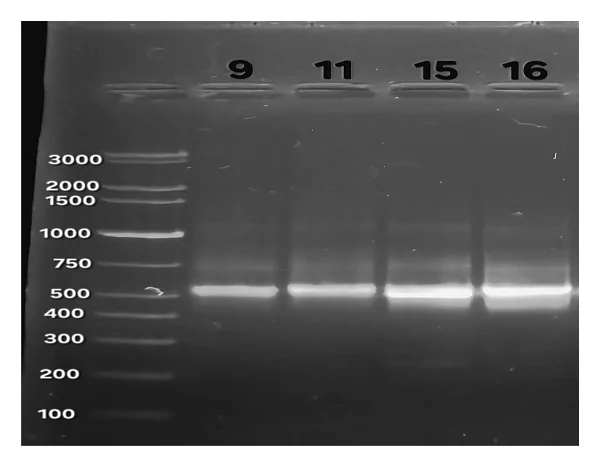
Agarose gel electrophoresis of the 16S rRNA gene (about 500 bp) for the four bacterial isolates, 1 kb: 1 kb DNA ladder.

After PCR amplification of the 16S rRNA gene of the four bacterial isolates using 16S rRNA primers, the amplification products (about 500 bp) were separated using agarose gel electrophoresis analysis (Figure 11), purified, and sequenced.

##### 3.9.2.1. Sequence Homology Analysis

###### 3.9.2.1.1. Sample No. 9

The resulting sequence of the 16 SR of Sample 9 was 455 bp. It was deposited in the NCBI database under Accession No. “PZ205634.” The similarity of the resulting sequences was determined using the BLASTn search of the NCBI GenBank database. The sequence homology of Isolate 9 showed a close homology to an uncultured bacterium clone (SY6_93) (Supporting Figure 4), exhibiting a 93.53% identity and a 97% query coverage. The low sequence identity indicates that the isolate cannot be categorized into a known genus and may represent a potentially novel bacterial lineage associated with a marine sponge.

**FIGURE 12:**
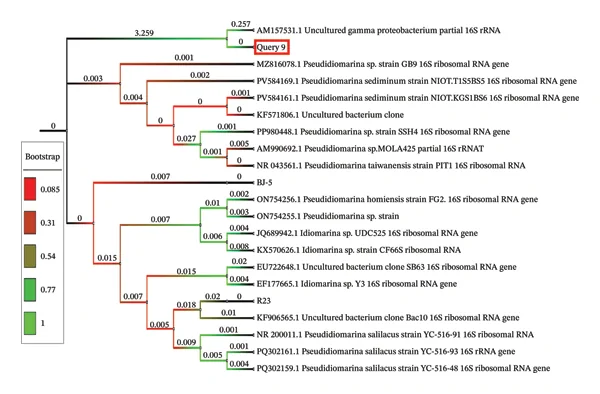
Phylogenetic tree of Isolate 9 (Query 9) including branch lengths and bootstrap values.

The phylogenetic analysis of the 16S rRNA gene indicated that the query sequence (Query 9) is a strong sister lineage to the uncultured *Gamma proteobacterium* sequence AM157531.1 (Figure 12). The green color of the node in the tree means that the bootstrap gave a lot of support to the connection between Query 9 and AM157531.1. This means that the two sequences have changed in very similar ways. This grouping shows that Query 9 is part of a Gammaproteobacterial lineage that is not well‐known and is very similar to a living marine taxon. These findings indicate that the isolate may signify a previously unrecognized taxon within the Gamma proteobacteria.

###### 3.9.2.1.2. Sample No. 11

**FIGURE 13:**
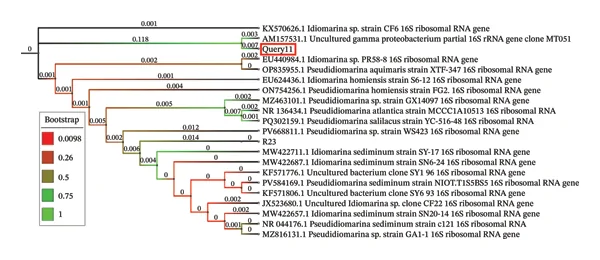
Phylogenetic tree of Isolate 11 (Query 11) including branch lengths and bootstrap values.

The 16 SR of sample 11 was 476 bp and was deposited in the NCBI database under Accession No. “PZ205862.” The BLASTn analysis of the partial 16S rRNA gene sequence from isolate 11 showed that it was most similar to an uncultured *Idiomarina* sp. clone CF22, with 83% query coverage. After that, closely related *Idiomarina*‐associated sequences showed up to 95.28% coverage (Supporting Figure 5). All of the top hits had very strong alignments (E‐value = 0.0), which means that they are very closely related to the genus *Idiomarina*. Even though BLASTn analysis showed that uncultured *Idiomarina* sp. clones had the most similar sequences, phylogenetic reconstruction showed that Query 11 was most closely related to uncultured Gamma proteobacterium AM157531.1 (Figure 13). This shows that local similarity searches are not always good for taxonomic inference and supports classification within an environmental Gamma proteobacterial lineage.

###### 3.9.2.1.3. Sample No. 15

**FIGURE 14:**
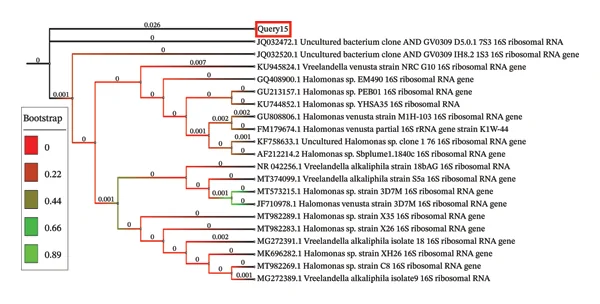
Phylogenetic tree of isolate 15 (Query 15), including branch lengths and bootstrap values.

The 16 SR sequence of sample 15 was 471 bp. It was deposited in the NCBI database under Accession No. “PZ205863.” The 16S rRNA gene sequence of Query 15 shows a high degree of similarity to several deposited sequences in GenBank, with a maximum BLASTn homology of about 98.54% (Supporting Figure 6) and very low E‐values (0.0), which means that it is very closely related to the genus *Halomonas*. The identity values, however, were below the species‐level threshold (around 98.7%–99% for 16S rRNA), which means that the isolate is likely a new species within the genus *Halomonas*. In the phylogenetic tree, Query 15 is part of this lineage and is surrounded by reference sequences that are very similar to it, not on its own long branch (Figure 14). The fact that BLASTn similarity and tree topology agree shows that the assignment is stable, at least at the genus level. In the genus *Halomonas*, Query 15 is best considered *Halomonas* species.

###### 3.9.2.1.4. Sample No. 16

The 16 SR sequence of sample 16 was 488 bp. It was deposited in the NCBI database under Accession No. “PZ205864.” The 16S rRNA gene sequence of Query 16 showed a very high level of homology (99.18%–99.78% identity, E‐value = 0.0) to several strains of *O. kapialis* when analyzed with BLASTn (Supporting Figure 7).

**FIGURE 15:**
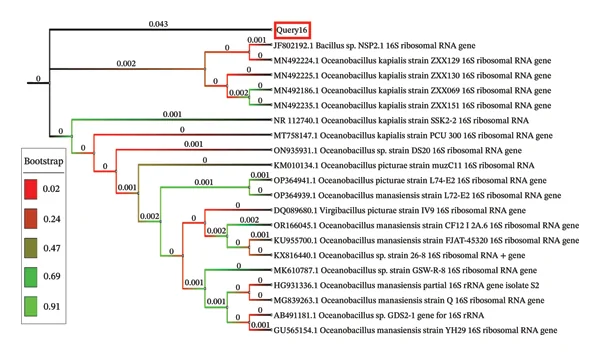
Phylogenetic tree of isolate 16 (Query 16) including branch lengths and bootstrap values.

In addition, the phylogenetic analysis using the 16S rRNA gene sequences unequivocally positioned Query 16 within the *Oceanobacillus* clade, specifically clustering with *O. kapialis* strains, as opposed to closely related species like *O. manasiensis* or *O. picturae* (Figure 15). The combined BLASTn and phylogenetic evidence strongly suggests that the isolate belongs to the genus *Oceanobacillus* and that it is *O. kapialis*. So, the best way to nominate Query 16 is *O. kapialis*.

## 4. Discussion

The successful separation of four morphologically distinct bacterial strains from *Ca. siphonella* tissues underscores the remarkable microbial diversity found in marine sponges. It confirms their status as plentiful reservoirs of biotechnologically relevant micro‐organisms. The four isolates have very different pigmentation patterns, from shiny orange (Sample 16) and light orange–brown (Sample 11) to golden‐yellow (Sample 9) and beige (Sample 15). This means that they make different secondary metabolites, especially carotenoids. Marine bacteria have been shown to use this method to deal with environmental stressors such as UV radiation, oxidative stress, and high salinity. Biotechnology has been very interested in marine bacteria that make carotenoids because they are antioxidants, antimicrobials, and protectors against UV light. People often connect golden‐yellow and orange pigments to β‐carotene and zeaxanthin derivatives [6, 41].

The granular texture and orange–brown color of Sample 11 strongly suggest that it is part of the *Actinomycete* group. This is consistent with reports indicating that members of this phylum frequently exhibit distinctive colony morphologies when cultivated on selective media [1, 41]. *Callyspongia* has been identified as a primary host for diverse bacterial communities, including Actinobacteria, Proteobacteria, and Firmicutes. Studies conducted in the Red Sea and South China Sea have revealed notable antimicrobial characteristics in these symbionts [42, 43]. The successful cultivation of pigmented bacteria from *Ca. siphonella* in this study, along with their subsequent use in the production of AgNPs, demonstrates the potential utility of marine sponge‐associated micro‐organisms for nanotechnology.

The successful bioreduction of silver ions to nanoparticles using marine bacterial cell‐free supernatants was evidenced by the temporal color change from pale yellow to light brown within 1 h and subsequently to dark brown over 24 h. This provides visual confirmation of the time‐dependent nature of the bioreduction process and directly correlates with the gradual accumulation of AgNPs. The rapid color change (within 1 h) indicates that the bacterial extracts possess potent reducing agents capable of converting Ag^+^ to Ag^0^ under mild conditions. These agents are likely proteins with natural reducing properties, flavonoids, polyphenols, and other bio‐organic compounds that act as both reducing and stabilizing agents at the same time. The color changed from light brown to dark brown over the course of 24 h, which shows that AgNPs kept forming and growing because the bacterial extract kept giving off reducing metabolites. This biphasic kinetic profile is consistent with classical nucleation theory and has been extensively documented in research on biogenic nanoparticle synthesis [10, 13].

The capping layer, made up of proteins, enzymes, and secondary metabolites from the marine bacterial extract, does a lot of important things, such as keeping nanoparticles from sticking together by using electrostatic forces to enhance colloidal stability, making nanoparticles less toxic by making them more compatible with living things, and making them more active by keeping bioactive surface compounds [13, 25]. This study’s green synthesis method is an example of sustainable nanomanufacturing that uses the natural biosynthetic abilities of marine bacteria. It shows a move toward nanotechnology that is better for the environment and follows the principles of green chemistry and a circular economy. These results collectively support the feasibility of enhancing marine bacteria‐mediated AgNP synthesis for biomedical and pharmaceutical applications, ensuring product sustainability and biocompatibility.

The comprehensive physicochemical characterization of the biosynthesized AgNPs using UV–visible spectroscopy, FTIR spectroscopy, TEM, and DLS provides definitive proof of the successful green synthesis and structural integrity of the nanomaterials. The optical signature of AgNPs is the characteristic SPR peak at about 460 nm that can be seen in all four AgNP samples. It also shows that the bioreduction process fully changed Ag^+^ ions into Ag^0^. The slight variations in the absorbance intensity among the four samples indicate that the concentrations and aggregation states of the nanoparticles differ, as each marine bacterial isolate possesses a distinct biochemical composition [26]. In addition, the red shift at 460 nm (which typically peaks between 400 and 420 nm) may reflect the local dielectric environment created by an organic biomolecular corona, particle–particle interactions, or a degree of aggregation/polydispersity [28].

The FTIR spectroscopic analysis provides molecular‐level confirmation of successful nanoparticle formation and biomolecule‐mediated synthesis. The fact that the biosynthesized AgNP samples had Ag–O and Ag–O–Ag stretching vibrations at 500–650 cm^−1^, which were not present in the silver nitrate control, is clear spectroscopic proof that the nanoparticles are made of silver. The bathochromic shift (red shift) of about 50 cm^−1^ seen in the fingerprint region (from 860/868 cm^−1^ in the blank to 810/806 cm^−1^ in AgNP samples) shows that the structure has changed and that the new nanoparticles are interacting with the biomolecular corona that comes from the bacterial extract. The alterations in the carbonyl and amide regions (1000–1800 cm^−1^), characterized by distinct peak patterns between the control and nanoparticle samples, indicate that functional groups from bacterial biomolecules (such as proteins, polyphenols, and secondary metabolites) participated in the reduction of silver ions and the capping of nanoparticles via surface coordination [30, 44].

TEM analysis revealed that all four bacterial isolate‐derived samples predominantly contained spherical nanoparticles with average diameters ranging from 7.28 to 13.8 nm. This size range is perfect for making antimicrobial agents work better. The fact that all the samples have the same spherical shape shows that the biomolecular reducing agents in the bacterial extracts helped control how quickly the nucleation and growth happened. The narrow size distribution in Samples 15 and 16, as opposed to the small aggregation in Samples 11 and 9, is likely due to the differences in the concentration and composition of capping biomolecules from each bacterial source. These differences change how nanoparticles stay stable and group together. The size range of 5–25 nm is positively correlated with the effective antimicrobial and cytotoxic activities demonstrated in subsequent biological assays, as nanoparticles within this dimensional range exhibit optimal surface area‐to‐volume ratios for cellular penetration and intracellular activity [45, 46].

These detailed characterization results show that high‐quality AgNPs can be made from marine bacterial symbionts of *Ca. siphonella* in a green way. This sets a strong foundation for future testing of these nanoparticles as therapeutic antimicrobial and promising anticancer agents.

Compared with representative AgNP systems, the four *Ca. siphonella*–associated bacterial formulations examined in the present study showed a consistent broad‐spectrum antibacterial profile, with TEM core sizes of 7.28–13.8 nm, modal hydrodynamic diameters of approximately 17–20 nm, inhibition zones of approximately 14–17 mm, MIC values of 8–16 μg/mL, and MBC values of 16–53.33 μg/mL against *S. aureus, E. coli, K. pneumoniae*, and *Ps. aeruginosa*. The previously reported values for biogenic AgNPs correspond with those for *Ps*. *aeruginosa*. This supports the idea that marine bacteria that live in symbiosis with other organisms can be used to make antimicrobial nanomaterials. AgNPs made from *Pseudomonas* sp. are interesting have been shown to have inhibition zones between 13 and 21 mm, which is found against similar pathogenic strains, with *P. aeruginosa* exhibiting a robust response to AgNPs treatment [47]. The increased susceptibility of Ps. The average inhibition zone of 16.34 ± 0.19 mm for aeruginosa in our study aligns with substantial literature demonstrating that Gram‐negative bacteria exhibit increased susceptibility to AgNPs owing to their thinner peptidoglycan cell walls, which promote nanoparticle infiltration and subsequent membrane disruption. Salomoni et al. [48] demonstrated that 10‐nm AgNPs eradicated approximately 99.9% of multidrug‐resistant *P. aeruginosa* hospital strains at a concentration of 5 μg/mL. They said this was because the particles made it easier for cells to touch each other and for silver to get into cells [47]. The antibacterial mechanism involves several pathways, such as disrupting membrane permeability, producing reactive oxygen species (ROS), interfering with respiratory enzymes, and inhibiting DNA replication. All of these pathways work together to kill bacteria, in the current study [49].

The closest ecological comparison identified was reported by Vasanthabharathi et al., who synthesized AgNPs using the cell‐free supernatant of *Ps. fluorescens* BCPBMS‐1 isolated from the marine sponge *Ca. diffusa*. Their formulation produced inhibition zones of approximately 5 mm against *E. coli*, 4 mm against *Proteus mirabilis* and *Salmonella typhi*, and 3 mm against *S. Paratyphi*, whereas no detectable inhibition was reported against *S. aureus* or *Vibrio cholerae*. In contrast, the present *Ca. siphonella*–associated AgNPs inhibited all four tested bacterial pathogens, including *S. aureus*, although differences in nanoparticle dose, well volume, bacterial strains, and assay conditions prevent a direct conclusion of superiority [50].

A more recent marine–bacterial system based on *Pl. maritimus* MBP‐2 generated AgNPs with an average TEM diameter of 24.9 nm and produced a maximum inhibition zone of 11 mm against *P. aeruginosa*, while no inhibition was observed against *S. aureus*; thus, the 15.83–17.00 mm zones obtained against *P. aeruginosa* and the reproducible activity against *S. aureus* in the present study indicate a comparatively broader apparent antibacterial spectrum under the respective test conditions [51].

Similarly, Bhatia et al. produced approximately 20‐nm PVP‐stabilized AgNPs using the supernatant of *Lysinibacillus boronitolerans* and reported inhibition zones of 13–22 mm, with MIC values of 15–20 μg/mL against *P. aeruginosa* and *S. aureus*. Although their maximum diffusion zone exceeded those observed here, the present formulations exhibited lower mass‐based MIC values against the corresponding organisms, namely, 8–13.33 μg/mL, illustrating that agar‐diffusion diameters and broth MIC values do not necessarily rank different nanoparticle formulations identically [52].

Compared with plant‐mediated synthesis, marine bacterial cell‐free supernatants offer a more controllable and reproducible platform for AgNP production because culture conditions, growth phase, and harvest time can be standardized. Marine bacteria also produce extracellular proteins, enzymes, polysaccharides, and metabolites that can act as reducing and capping agents and influence nanoparticle size, stability, and antimicrobial activity. This approach has been successfully demonstrated using marine bacteria such as *Nocardiopsis dassonvillei* and *Planococcus maritimus* [53]. In contrast, plant extracts are simpler, faster, and less expensive, but their composition may vary with plant species, tissue, season, and extraction method, leading to differences in nanoparticle properties [54]. Therefore, marine bacterial and plant‐mediated synthesis should be considered complementary green approaches, with the bacterial route offering greater process control but requiring sterile cultivation and careful purification [55].

The absence of detectable antifungal activity against *C. albicans* in our study presents an intriguing finding that contradicts several reports demonstrating the efficacy of AgNPs against fungal pathogens [56–58]. AlJindan et al. [59] recorded significant antifungal effectiveness of AgNPs against *C. auris*, with MIC values below 6.25 μg/mL [59]. The lack of antifungal activity in our samples may be attributed to the distinctive physicochemical properties imparted by the marine bacterial capping agents, including particle size distribution, surface charge, and corona composition, which collectively influence cellular interactions and antimicrobial selectivity [60]. This specific antibacterial activity with diminished antifungal effects may represent a therapeutically advantageous attribute. Curcumin‐stabilized AgNPs exhibited improved stability and selective toxicity to bacteria over mammalian cells, highlighting the potential for biocompatible antimicrobial applications [61]. Khorrami et al. [62] demonstrated the selective cytotoxicity of green‐synthesized AgNPs, indicating that biosynthesized AgNPs caused only 15% cell death in normal L‐929 fibroblast cells at a concentration of 60 μg/mL after 48 h, while simultaneously exhibiting significant antibacterial activity against both Gram‐negative and Gram‐positive bacteria at MICs of 5–30 μg/mL [62]. Wen et al. [63] investigated the antifungal mechanisms of AgNPs on *Ustilaginoidea virens*, demonstrating that AgNPs induce membrane disruption in fungal cells through pathways analogous to mammalian cell toxicity, including enhanced permeability and endocytosis, resulting in intracellular damage. The study rigorously established that fungal cell membranes react to AgNPs via mechanisms similar to those observed in eukaryotic cells [63]. As a result, there is no antifungal activity against *Candida*. The presence of albicans in our study, coupled with the demonstrated antibacterial efficacy, suggests that these marine‐derived AgNPs may possess favorable selectivity profiles with reduced cross‐toxicity toward eukaryotic cells, including human cells that exhibit structural similarities with fungal membranes. This hypothesis requires additional investigation via comprehensive cytotoxicity and selectivity index analyses.

The quantitative evaluation of antimicrobial efficacy via MIC determination indicated significant disparities in susceptibility profiles among the four bacterial species and between the various marine bacteria‐derived AgNP samples. The MIC of all four AgNP samples against *E. coli* was about 8 μg/mL. *E. coli*. The results concerning *E. coli* are particularly significant and closely correspond with established literature values for biogenic AgNPs. A MIC of 7.5 μg/mL was specifically reported for marine‐derived AgNPs against *E. coli* [64], and a MIC of 4 μg/mL for green‐synthesized AgNPs against the same pathogen, corroborating the reliability and effectiveness of our results [65]. The susceptibility in all samples indicates that the standardized particle size range (7.28–13.8 nm) and surface chemistry of our biosynthesized nanoparticles are ideally configured for infiltrating Gram‐negative bacterial cell membranes, given that this organism typically features a thin peptidoglycan layer and an outer membrane structure conducive to nanoparticle internalization.

The four samples showed different levels of antimicrobial effectiveness, with AgNPs‐15 and AgNPs‐16 having lower MIC values (8–13.33 μg/mL) against *S. aureus* and better activity against Ps. The differences between AgNPs‐9 and AgNPs‐11 and aeruginosa show that the physicochemical properties of the nanoparticles are slightly different. This is probably because each marine bacterial isolate has a different biochemical makeup and ability to reduce. The hierarchical ordering of mean particle diameters—AgNPs‐15 (8.89 nm) < AgNPs‐16 (9.39 nm) < AgNPs‐9 (10.68 nm) < AgNPs‐11 (12.43 nm)—is directly related to their different antimicrobial activity. The biogenic AgNPs made at different pH levels had MIC values that ranged from 0.125 to 1 μg/mL, depending on the size and surface charge of the particles. Smaller nanoparticles were more effective at killing bacteria [66].

The differential susceptibility pattern, with *E. coli* had low MIC values that were the same for all samples, while *S. aureus, K. pneumoniae,* and *Ps. aeruginosa* displayed more variable MIC profiles, aligning with previously recorded differential responses of Gram‐positive and Gram‐negative bacteria to AgNPs. Khalil et al. [53] found that *Ps. aeruginosa* had the lowest MIC of bioengineered AgNPs at 4 μg/mL. This is because this Gram‐negative pathogen is known to be very resistant to oxidative stress [53]. Conversely, a MIC of 6.5 μg/mL for *S. aureus* against marine‐derived AgNPs was reported [67], slightly lower than most of our values (8–13.33 μg/mL), possibly indicating variations in nanoparticle size or surface modifications [64]. The MIC values for K. Our study’s findings on the susceptibility of pneumoniae (8–16 μg/mL) to marine bacteria‐derived AgNPs constitute one of the initial comprehensive analyses of this increasingly multidrug‐resistant pathogen, laying the groundwork for subsequent comparative studies.

The analysis of bactericidal activity showed that the MBC values were very different for both the four marine bacteria‐derived nanoparticle samples and the tested bacterial species. This gave us important information about how specific and powerful these biosynthesized antimicrobial agents are. The MBC/MIC ratios, which range from 2.0 to 4.0 for the samples and species, show that these AgNPs are mostly bactericidal, which is an important difference between them and bacteriostatic antimicrobial agents.

Parvekar et al. [34] recorded MBC values of 0.625 mg/mL for 5 nm chemically synthesized AgNPs against *S. aureus,* which is much lower than our biogenic AgNPs (21.33 ± 5.33 μg/mL) when compared to literature values [34]. This difference is probably because our marine bacteria‐derived nanoparticles have a wider range of particle sizes than the 5‐nm particles used by Parvekar et al. [34]. Siddique et al. [67] reported MBC values ranging from 250 to 500 μg/mL for chemically synthesized AgNPs against multidrug‐resistant K. pneumoniae strains [67], which are much higher than our biogenic AgNPs. This is probably because the MDR sample needs a higher concentration of AgNPs. MBC values of 3.246 ± 1.056 μg/mL for chemically synthesized AgNPs against multidrug‐resistant *Ps. aeruginosa*, which is significantly lower than the values for our biogenic AgNPs (26.67–32 ± 0.0–5.33 μg/mL) [49].

The concentration‐dependent cytotoxicity exhibited by the four marine bacteria‐derived AgNPs against rat cancer cells signifies a promising discovery for their potential use as anticancer nanotherapeutics. The slight inhibition seen at the lowest concentration (10 μg/mL), which was between 10% and 20%, shows that a certain nanoparticle concentration is needed for effective cytotoxic activity. This is different from the lower MIC values needed for antibacterial activity (8–16 μg/mL), which suggests that prokaryotic and eukaryotic cancer cells respond differently. The IC50 values for all four samples are very close to each other, between 42 and 44 μg/mL. This shows that the cytotoxic potency is very consistent, even though the samples came from different bacterial isolates. The IC50 values are very similar, with only a 2 μg/mL difference (42–44 μg/mL). This suggests that the size range of the nanoparticles (7.28–13.8 nm) and their physicochemical properties were the main factors that determined their cytotoxic activity. The differences in reducing agents between bacterial isolates had little effect on the final cytotoxic phenotype.

The resulting IC50 values of 42–44 μg/mL in the current study fall within the moderate range of biogenic AgNPs documented in the literature. For comparison, Ahmad et al. [68] reported IC50 of 38.13 μg/mL for green‐synthesized AgNPs against lung cancer A‐549 cells [68], while Khaled et al. [68] documented IC50 of 1.16 μg/mL for berberine‐loaded AgNPs against hepatocellular carcinoma HepG2 cells, indicating that drug‐loaded nanoparticles achieve substantially lower IC50 values. The agreement between microscopic phenotype and *16S rRNA* identification strengthens the taxonomic assignment of our isolates: All four taxa have typical morphologies that match their molecular placements. Members of the class Gammaproteobacteria are uniformly Gram‐negative and morphologically diverse (rods, curved rods, coccoid forms) [14], so the observation of Gram‐negative rods or slightly curved rods is broadly consistent with assignment to this class. Specifically, isolates classified as *Idiomarina* displayed the characteristic Gram‐negative, slightly curved rod morphology, accompanied by motility indicative of *Monotrichous flagellation* [69]. The *O. kapialis* isolate exhibited Gram‐positive, rod‐shaped, spore‐forming morphology, characteristic of the genus *Oceanobacillus* [70]. Taken together, the microscopy and Gram‐stain images provide independent phenotypic validation of the *16S rRNA* results. This combined phenotypic–genotypic approach follows best practices for bacterial identification, improving confidence where 16S resolution alone can be ambiguous, and is particularly important for environmental and sponge‐associated isolates that may represent novel or poorly sampled lineages.

The reported antimicrobial and cytotoxic activity of related bacterial isolates in the current study documented that an *Idiomarina* strain (OM679414.1) was shown to produce fatty‐acid metabolites that inhibited both Gram‐positive and Gram‐negative pathogens and killed ∼91% of SK‐MEL‐3 melanoma cells at ∼15 μg/mL [16]. In addition, extracts and AgNPs from marine *Halomonas* inhibited *S. aureus*, *P. aeruginosa,* and *C. albicans* (zones ∼11–12 mm, MIC ∼128–256 μg/mL) [17], and *H. saccharevitans* extracts showed significant cytotoxicity against HepG2 liver cancer cells [18].

This study’s exploratory design, which employs a limited experimental framework to assess the biological potential of biosynthesized AgNPs. The results derived from a small number of bacterial isolates restrict generalizability, despite the nanoparticles showing promising antibacterial and potential anticancer properties. Additional characterization, including zeta potential and long‐term stability, is essential. The anticancer evaluation was confined to short‐term in vitro cytotoxicity without considering normal cells or apoptotic processes; therefore, future studies are needed using validated assays such as MTT, XTT, or CCK‐8 after 24, 48, and 72 h of exposure, paired human cancer and nontumoral cell lines, and mechanistic analyses of cell death. Furthermore, comprehensive genomic and metabolomic investigations are necessary to identify the biomolecules involved in the nanoparticles′ actions. Future research should aim to diversify isolates, expand pathogen and cancer cell panels, incorporate normal cell controls, and validate efficacy in vivo to thoroughly characterize the therapeutic potential of these marine bacteria‐derived AgNPs.

This study highlights the efficacy of marine sponge‐associated bacteria, specifically *Gammaproteobacterium*, *Idiomarina* sp., *Halomonas* sp., and *O. kapialis*, in the green synthesis of AgNPs with significant biomedical applications. Strong correlations between phenotypic traits and *16S rRNA* identification confirm reliable taxonomic classifications, advocating for combined molecular and microscopic methods in environmental studies. The biosynthesized AgNPs demonstrated potent antibacterial activity at low MIC values and moderate cytotoxic effects, influenced by nanoparticle size and surface chemistry. Notably, they showed no antifungal activity against *C. albicans*, indicating a selective profile that may limit toxicity to eukaryotic cells. The research underscores the potential of sponge‐associated bacteria as sustainable biofactories for biogenic nanoparticles and sets the stage for optimizing these materials for antimicrobial use. The four studied taxa exhibit halotolerance and a strong secondary metabolism, positioning them as promising sources for new antimicrobial agents and integral to the chemical defenses of their sponge hosts.

## Funding

No funding was received for this manuscript.

## Conflicts of Interest

The authors declare no conflicts of interest.

## Supporting Information

Additional supporting information can be found online in the Supporting Information section.

## Supporting information


**Supporting Information 1** Supporting Figure 1. MIC determination by broth microdilution in 96‐well plates. Green rectangles around wells indicate ‐ve controls, while red ones indicate + ve controls. Red circles in each row represent the MIC well. Rows A, B, C, and D, and then E, F, G, and H correspond to *S. aureus, E. coli, K. pneumonia,* and *Ps. aeruginosa,* respectively, for two samples in each plate (15 and 16 in Plate A, 9 and 11 in Plate B). This step was performed in triplicate, so some MIC results in the figure may differ from the mean results in Table 3.

**Supporting Information 2** Supporting Figure 2. Some MBC results are shown in MHA plates; A, B: plates for AgNPs‐16 show MBC against *S. aureus, E. coli,* and *K. pneumonia*, C: AgNPs‐11 shows MBC against *S. aureus, E. coli,* and *K. pneumonia*, D: AgNPs‐9 show MBC against *E. coli,* and *K. pneumonia*, E, F: and plates on the right top and bottom for AgNPs‐15 show MBC against *S. aureus, E. coli, K. pneumonia,* and *Ps. aeruginosa*. Red circles represent all MBC results in the plates. Numbers with the letters represent the concentration of AgNPs.

**Supporting Information 3** Supporting Figure 3. Gram staining technique of four bacterial isolates: 15, 11 are Gram‐negative, and 9 and 16 are Gram‐positive.

**Supporting Information 4** Supporting Figure 4. Results of BLASTn alignment for Isolate 9.

**Supporting Information 5** Supporting Figure 5. Results of BLASTn alignment for Isolate 11.

**Supporting Information 6** Supporting Figure 6. Results of BLASTn alignment for Isolate 15.

**Supporting Information 7** Supporting Figure 7. Results of BLASTn alignment for Isolate 16.

## Data Availability

The data that support the findings of this study are available from the corresponding author. The DNA sequences were deposited in the NCBI GenBank as follows: Sample 11 (PZ205862): https://www.ncbi.nlm.nih.gov/nuccore/PZ205862.1?report=GenBank. Sample 15 (PZ205863): https://www.ncbi.nlm.nih.gov/nuccore/PZ205863.1?report=GenBank. Sample 16 (PZ205864): https://www.ncbi.nlm.nih.gov/nuccore/PZ205864.1?report=GenBank. Sample 9 (PZ205634): https://www.ncbi.nlm.nih.gov/nuccore/PZ205634.1?report=GenBank.
