# Supplementary material for: The Antimicrobial and Cytotoxic Activity of Green‐Synthesized Silver Nanoparticle Extract of Marine Bacteria and Their 16S rRNA Identification and Characterization
Source: Bioinorg Chem Appl. 2026 Sep 20;2026:9040709. doi: 10.1155/bca/9040709 (PMC13591115; doi:10.1155/bca/9040709)
Supplement: Supplementary file 1 [file BCA-2026-9040709-s001.docx]

**D**

**B**

**A**Antimicrobial activity of silver nanoparticles samples (AgNPs 9, AgNPs 11, AgNPs 15, and AgNPs 16) and mixtures between them (15mix and 16 mix) observed by inhibition zones using well diffusion method. Negative controls of bacterial extract show no inhibition zones while AgNo3 control show small inhibition zone in some plates. All AgNPs samples show inhibition zones against four types of bacteria in different sizes (st on the plate on the top left; *S. aureus*, E on the middle top; *E. coli*, Ps on the right top; *Ps. aeruginosa*, and K on the bottom left plate; *K. pneumonia*) while they didn’t show antifungal activity (C on the right bottom; *Candida albicans)*.

**F**

**E**

**C**

**Supplementary Figure 2.** Some MBC results shown in Mueller Hinton agar plates; A,B: plates for AgNPs-16 show MBC against *S. aureus, E. coli,* and *K. pneumonia*, C: AgNPs-11 shows MBC against *S. aureus, E. coli,* and *K. pneumonia*, D: AgNPs-9 show MBC against *E. coli,* and *K. pneumonia*, E,F: and plates on the right top and bottom for AgNPs-15 show MBC against *S. aureus, E. coli,* *K. pneumonia,* and *Ps. aeruginosa*. Red circles represent all MBC results in the plates. Numbers with the letters represent the concentration of AgNPs.
