# Supplementary material for: The Antimicrobial and Cytotoxic Activity of Green‐Synthesized Silver Nanoparticle Extract of Marine Bacteria and Their 16S rRNA Identification and Characterization
Source: Bioinorg Chem Appl. 2026 Sep 20;2026:9040709. doi: 10.1155/bca/9040709 (PMC13591115; doi:10.1155/bca/9040709)
Supplement: Supplementary file 2 [file BCA-2026-9040709-s002.docx]

.

**11**

**16**

**9**

**15**

**Supplementary Figure 3.** Gram staining of the four bacterial isolates: 15, 11 are Gram-negative, and 9 and 16 are Gram-positive.
