# Supplementary material for: The Antimicrobial and Cytotoxic Activity of Green‐Synthesized Silver Nanoparticle Extract of Marine Bacteria and Their 16S rRNA Identification and Characterization
Source: Bioinorg Chem Appl. 2026 Sep 20;2026:9040709. doi: 10.1155/bca/9040709 (PMC13591115; doi:10.1155/bca/9040709)
Supplement: Supplementary file 6 [file BCA-2026-9040709-s006.docx]

**Dilution (µg/mL)	256	128	64	32	16	8	4	2	1	0.5	0.25 0.12**

**16**

**16**

**16**

**16**

**15**

**15**

**15**

**15**

**A**

**Dilution (µg/mL)	256	128	64	32	16	8	4	2	1	0.5	0.25 0.12**

**9**

**9**

**9**

**9**

**11**

**11**

**11**

**11**

**B**

**Supplementary Figure 1.** MICs determination by broth microdilution in 96-well plates. Green rectangles around wells indicate -ve controls, while red ones indicate +ve controls. Red circles in each row represent the MIC well. The rows A, B, C, and D, then E, F, G, and H correspond to *S. aureus, E. coli, K. pneumonia, and Ps. aeruginosa,* respectively, for two samples in each plate (15 and 16 in plate A, 9 and 11 in plate B). This step was performed in triplicate, so some MIC results in the figure may differ from the mean results in Table 3.
