# Supplementary material for: The Antimicrobial and Cytotoxic Activity of Green‐Synthesized Silver Nanoparticle Extract of Marine Bacteria and Their 16S rRNA Identification and Characterization
Source: Bioinorg Chem Appl. 2026 Sep 20;2026:9040709. doi: 10.1155/bca/9040709 (PMC13591115; doi:10.1155/bca/9040709)
Supplement: Supplementary file 8 [file BCA-2026-9040709-s004.docx]

**Supplementary** **Figure 6.** Results of BLASTn alignment for isolate 15.
